# Editorial: Identification of key factors to improve performance in Olympic water sports

**DOI:** 10.3389/fspor.2024.1388344

**Published:** 2024-03-05

**Authors:** Catarina C. Santos, Francisco Cuenca-Fernández, Ricardo J. Fernandes, Mário J. Costa

**Affiliations:** ^1^ Centre of Research, Education, Innovation and Intervention in Sport (CIFI2D), Faculty of Sport, University of Porto, Porto, Portugal; ^2^ Department of Sport Sciences, Higher Institute of Educational Sciences of the Douro (ISCE-Douro), Penafiel, Portugal; ^3^ Department of Sports and Computer Sciences, University of Pablo de Olavide, Seville, Spain; ^4^ Aquatics Lab, Department of Physical Education and Sports, Faculty of Sport Sciences, University of Granada, Granada, Spain; ^5^ Porto Biomechanics Laboratory (LABIOMEP-UP), University of Porto, Porto, Portugal

**Keywords:** swimming, water polo, canoeing, assessment, training

**Editorial on the Research Topic** Identification of key factors to improve performance in Olympic water sports

Water sports can be categorized into team or individual sports and can be performed in different aquatic environments (e.g., swimming pool, river or sea). Among those, swimming, open water swimming, artistic swimming, diving, water polo, rowing, canoeing (canoe/kayak), sailing and surf are recognized as Olympic sports. Although they share the same approach from a scientific analysis standpoint (e.g., biomechanical, physiological and anthropometrics), they differ on which are the most specific and determinant factors for winning. Within this, the Research Topic entitled *Identification of key factors to improve performance in Olympic water sports* aimed to gather scientific knowledge of Olympic water sports regarding: (i) biophysical factors that determine performance; (ii) acute and long-term effects on performance determinant factors; (iii) the use of different equipment and technologies to maximize the training effects; (iv) valid and reliable methodologies for testing and training control (including feedback); and (v) performance pathways or long-term athletic development strategies.

**Figure 1:**
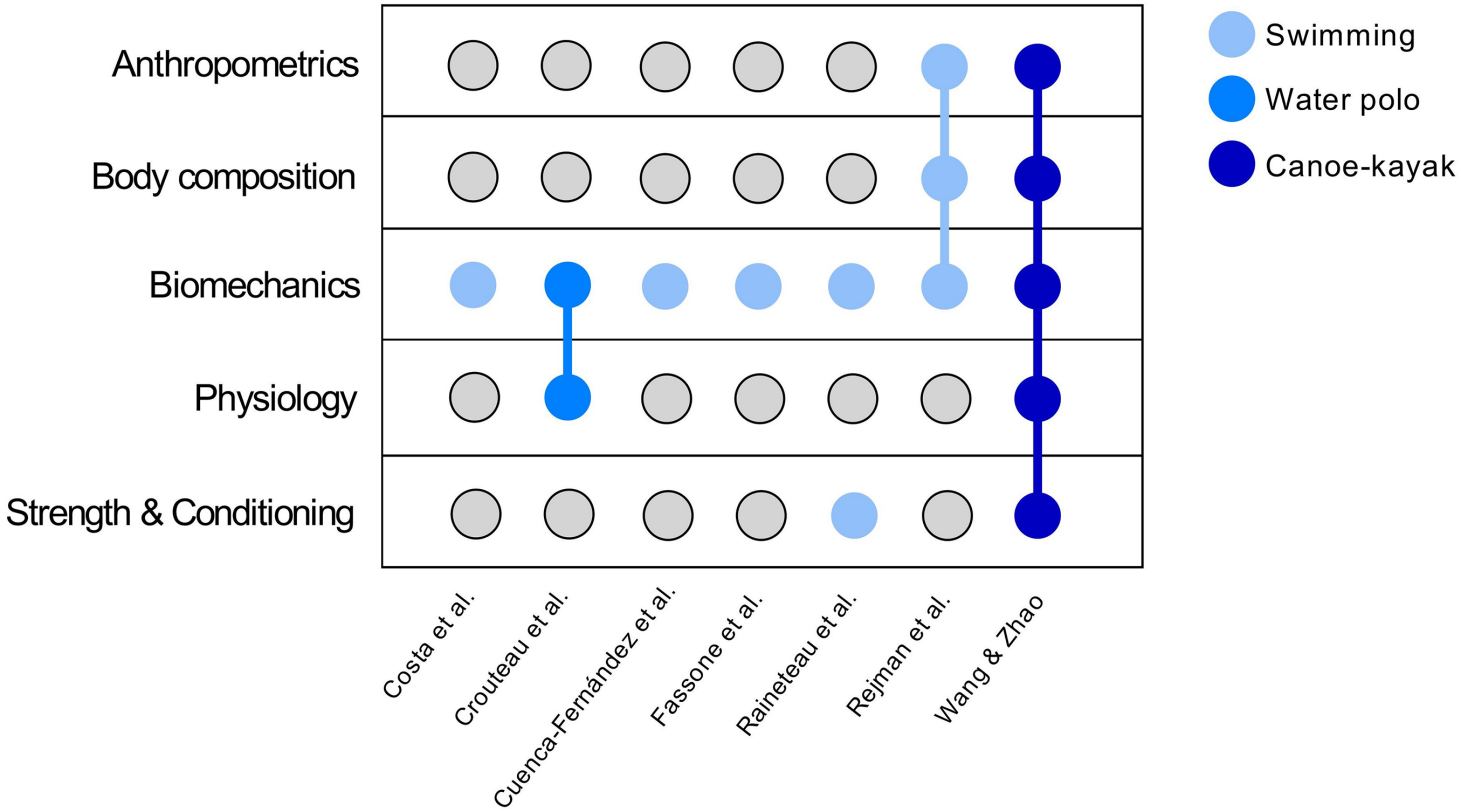
The main areas of interest for each study (*n* = 7).

Seven articles were included in this Research Topic, being six published in Frontiers in Sports and Active Living (Costa et al., Croteau et al., Cuenca-Fernández et al., Fassone et al., Raineteau et al. and Rejman et al.) and one in Frontiers in Physiology (Wang and Zhao). The main areas of interest within these studies were anthropometrics, body composition, biomechanics, physiology and strength & conditioning distributed for swimming, water polo and canoeing (Figure 1). Considering the first author demographics of each study, most articles had origin in Europe (71.4%), followed by North America (14.3%) and Asia (14.3%).

Competitive swimming from childhood to adulthood aims to accomplish a given distance in the shortest time (1), the reason why researchers try to understand the way to excel within the major competitions by considering the progression and variability between and within events (2, 3). One study aimed to analyze the performance variation in all rounds and sections of the male elite 100 m and 200 m events and to determine the performance level effect in the final outcome (Cuenca-Fernández et al.). Swimmers who competed at the LEN 2019 European Short Course Swimming were analyzed, being observed that: (i) 100 m finalists progressed between rounds mainly due to the improvement in start and split times during lap 1; (ii) higher performance maintenance was observed in the round-to-round analysis of 200 m than 100 m events; and (iii) only backstroke finalists showed lower variation in split times compared to non-qualified swimmers. Despite concluding that the finalist's success can be attributed to overall higher performance levels and progression, it was assumed that elite performances are often composed by outliers.

It is well known that competitive swimming is based on a multifactorial and dynamic phenomenon, where anthropometrics and biomechanical domains define the energetic profile and can contribute to performance enhancement (4). Within this, the relationships with the force output and performance have been a topic of interest in recent years (5). While assessing the associations between force production (tethered-swimming) and 100 m front crawl inter-lap pacing and kinematics in 11 elite male swimmers, Costa et al., found an increase in lap time along with a decrease in velocity, stroke rate and stroke index between the two 50 m laps, while stroke length remained unchanged. They also found a positive association between peak force and the ability to maintain a stable swimming velocity between 100 m front crawl laps, even if both pacing and kinematics fall from the first to the second 50 m sections. In the same line, Croteau et al., attempted to understand the associations between load-velocity profiles (semi-tethered swimming), kinematics and performance in eight elite male swimmers who performed the 100 m and 200 m front crawl. The estimated maximal velocity did not determine the studied performances but was strongly related to kinematics (stroke rate, stroke length and stroke index). However, when the load was normalized to the body mass, a strong correlation with the performance on the 100 m was found. So, it was emphasized that the relationships between the analyzed profiles, kinematics and performance depend on the event distance.

Regarding stroke length monitoring, Fassone et al., aimed to develop a methodology mostly based on metronome daily use. Data underscored the importance of using a metronome throughout the training season to control young swimmers stroke length changes, since results revealed that values were higher during the in-season comparing to pre-season. On the other hand, Rejman et al., explored which somatic features are common to the four competitive swimming techniques and medley, with the allometric model run in 130 swimmers revealing that there were five common swimming speed predictors: age^2^, upper body circumference, handbreadth, waist circumference and subscapular skinfold thickness. So, authors stated that results reinforced the idea that swimming performance underlies a “V-shape” trunk, longer upper limbs and large hands.

As the goalkeeper is determinant during the water polo teams' defensive stance (6), Croteau et al., monitored three female senior goalkeepers from the Canadian water polo national team. Players used an inertial measurement unit to measure the external load of lower limbs actions, jumps and player-load, while the internal load was defined as the session rating of perceived exertion. From 155 sessions, it was concluded that internal and external load can be monitored using self-reported and inertial devices measures, but it should be noted that former measures can differ across the goalkeepers within the same session, while latter measures differ across training contexts. In another specific Olympic water sport, Wang and Zhao compared a set of measures (Figure 1) in sprint kayakers according to different age groups and expertise levels, being observed that speed, physiologic indicators, strength and muscle mass were the main measures that distinguish the different age groups and expertise levels. Authors also found that international-level sprint kayakers exhibited better when compared to club-level athletes.

Since today's winners reach the podium with a small advantage, every factor that can determine better performances should be regularly tested. Accordingly, the current Research Topic gathered novel information on how coaches and researchers can help top-level athletes within the spectrum of Olympic water sports to become better while pursuing their goals.
